# Exploring the Views of Healthcare Professionals Working in a Mental Health Trust on Pharmacists as Future Approved Clinicians

**DOI:** 10.3390/pharmacy10040080

**Published:** 2022-07-12

**Authors:** Balazs Adam, Richard N. Keers

**Affiliations:** 1 Centre for Pharmacoepidemiology and Drug Safety, Division of Pharmacy and Optometry, The University of Manchester, Manchester M13 9PL, UK; richard.keers@manchester.ac.uk; 2 Pharmacy Department, Surrey and Borders Partnership NHS Foundation Trust, Leatherhead KT22 7AD, UK; 3 Suicide, Risk and Safety Research Unit, Greater Manchester Mental Health NHS Foundation Trust, Manchester M25 3BL, UK

**Keywords:** mental health pharmacy, responsible clinician, mental health policy, pharmacy education

## Abstract

This qualitative research explored the views and attitudes of healthcare professionals towards the role of the mental health pharmacist, and whether this group should be enabled to become approved clinicians (ACs) in England and Wales under the Mental Health Act in future. Following ethical approval, recruitment based on systematic purposive sampling principles took place at one mental health trust in England. Six pharmacists, five medical ACs and two mental health nurses participated in one-to-one digitally audio-recorded semi-structured interviews between June and November 2020. The recordings were transcribed verbatim before being inductively coded and thematically analysed. Notwithstanding the wide recognition among participants of several key skills possessed by mental health pharmacists, various obstacles were identified to them becoming ACs in future, including prevalent conventional models of pharmacy services delivery restricting adequate patient access, as well as insufficient training opportunities to acquire advanced clinical skills, particularly in diagnosis and assessment. In addition to the inherent legislative hurdles, fundamental changes to the skill mix within multidisciplinary mental health teams and improvements to the training of pharmacists were reported by participants to be required to equip them with essential skills to facilitate their transition towards the AC role in future. Further research is needed to gain a better understanding of the challenges facing the clinical development and enhanced utilisation of mental health pharmacists and non-medical ACs across services.

## 1. Introduction

### 1.1. Approved Clinicians

The Mental Health Act (MHA) 1983 is the predominant statutory legislation with respect to the powers and lawful processes governing the detention and treatment of people diagnosed with a mental disorder in England and Wales. The 2007 amendment to the MHA instituted several key changes concerning the responsibilities and powers of professionals and introduced the roles of approved mental health professionals (AMHPs)—who, traditionally, had been social workers only—and the responsible clinician (RC)—formerly medical doctors only. Simultaneously, both roles became accessible to several additional non-medical professionals that were previously not considered by the Act—because, as it was asserted at the time, “leadership … should be based primarily on competence rather than profession” [1]. This meant that henceforth medical doctors, social workers, mental health and learning disability nurses, chartered psychologists and occupational therapists could all take up the role of the AMHP or the RC [2,3,4].

Since the 2007 amendment, the list of professionals permitted to qualify and lawfully exercise these powers has remained unchanged.

Of these two roles, the RC is the professional in charge of taking overall responsibility for directing all aspects of patient care, including treatment, release, discharge, as well as community treatment orders (CTOs). As set out by the MHA 2007, before one may become a particular patient’s RC, they are first required to obtain the professional status of approved clinician (AC).

Whilst the MHA permits a range of professionals to qualify as an AC, there remain sharp distinctions between those from a medical and non-medical background, which are summarised in Box A1.

There are also pronounced differences in the numbers of medical and non-medical ACs. According to the first national survey conducted in England and Wales in 2017, only 56 non-medical ACs had been appointed, which is in stark contrast with over 6000 medical ACs [4]. This study highlighted several reasons for this discrepancy, including the training route to becoming a non-medical AC being unclear and laborious for candidates in comparison to medical colleagues. A previous survey by Ebrahim identified additional contributing factors to this difference in numbers, such as a lack of protected clinical time allocated to training by employers [5].

### 1.2. Mental Health Pharmacists

Over the years, the combination of purposeful integration of evidence-based practice and increased complexity of drug therapy has given birth to the concept of clinical pharmacy, and this has meant that increasingly pharmacists have been released from the logistics of the dispensing process with the help of new technologies and an improved skill mix. These changes have not only facilitated pharmacists’ progression towards establishing oversight of medicines management [6,7] but also contributed to the creation of the more holistic, patient-centred practice of medicine optimisation [8]. The introduction of non-medical prescribing (NMP)—the practice of prescribing medicines by healthcare professionals other than doctors—has not only enabled improved access to medicines but also further enhanced the management of medicines as well as patient safety [9]. For example, a study by Turner, Kennedy and Barrowcliffe conducted at a large National Health Service (NHS) acute hospital in the UK found that pharmacists made significantly fewer prescribing errors than doctors [10]. The UK College of Mental Health Pharmacy (CMHP) and the Department of Health have jointly stated that individuals with mental health problems are particularly suitable for management by non-medical prescribers [11]. Furthermore, patients often particularly value pharmacists’ knowledge and appreciate them promoting shared decision-making to a greater extent [11]. There is also increasing evidence for the use of pharmacists to support the management of patients with mild to moderate mental health conditions in primary care [12]. Harms et al. have demonstrated that clinical pharmacists can improve common primary outcomes associated with such conditions whilst also advocating interdisciplinary collaboration, improving standards of documentation and necessitating regular follow-up [13]. Several prior studies have also highlighted the unexploited potential of specialist mental health pharmacists within a multidisciplinary environment [14,15,16,17,18,19], whilst other more recent works have further demonstrated the value of their clinical interventions [20,21,22].

In spite of the aforementioned progressive transformations of pharmacy services leading to pharmacists assuming increasingly patient-facing and clinical roles, as of 2022, the MHA has not been reviewed with respect to considering this group of professionals for the role of the non-medical AC. Furthermore, the role of pharmacists has thus far not been evaluated in the published literature in relation to assuming the role of the AC. The aim of this qualitative research was therefore to explore, in the context of current non-medical AC role provision and service delivery challenges, acceptability toward and feasibility of the potential inclusion of mental health pharmacists as non-medical ACs in future.

## 2. Materials and Methods

### 2.1. Study Setting and Recruitment

For the purpose of recruiting participants for this study, the method of systematic purposive sampling was selected. All candidates who took part were invited via the host NHS trust’s internal email and provided with the participant information sheet and consent form. All completed consent forms were received via email prior to the interviews taking place.

The hosting NHS trust is regarded as a leading provider of health and social care services, employing approximately 2400 members of staff. Services are delivered in both inpatient and community settings and include mental health services for adults as well as children and adolescents, drug and alcohol and eating disorders, just to name a few.

There were no non-medical ACs employed by the trust at the time of the research, and this impacted on the selection criteria. Thus, eligibility criteria were determined based upon candidates’ respective professional affiliation as follows: (1) medical ACs, (2) mental health pharmacists, and (3) other mental health professionals of notable relevant experience—that is, any individual employed at Agenda for Change (AfC) (the NHS terms and conditions of service) [23] band 8 or above (typically these are skilled professionals in managerial and/or clinical leadership positions), preferably those currently working towards the AC qualification or having significant expertise in the subject. The sizes of the sample populations the researchers targeted were not available to the research team.

An overall target sample size of 10–15 was determined to both be feasible for this study—considering resource limitations—and sufficient to yield meaningful results. In addition, this sample size was also anticipated to be large enough to achieve data saturation. These predictions were based upon preliminary informal discussions with potential candidates within the researcher’s own professional network.

As the research question itself was centred around pharmacists, thoroughly exploring their own unique views was imperative to gaining sufficient insights in order to allow practical conclusions to be drawn. Since the study also focused extensively on the AC role, recruiting qualified ACs in high enough numbers was deemed imperative. These two considerations played an important role in determining the relative sizes of participant groups 1 and 2.

### 2.2. Data Collection

Each participant attended one semi-structured interview with the researcher (B.A.), with each designed to last approximately one hour in duration. Every candidate invited attended the interview and no participant was later removed from the study. Most of the participants shared a working relationship with B.A. The interviews took place between June and November 2020; some were conducted in person on trust premises while others were held virtually via Microsoft Teams.

**Figure A1:**
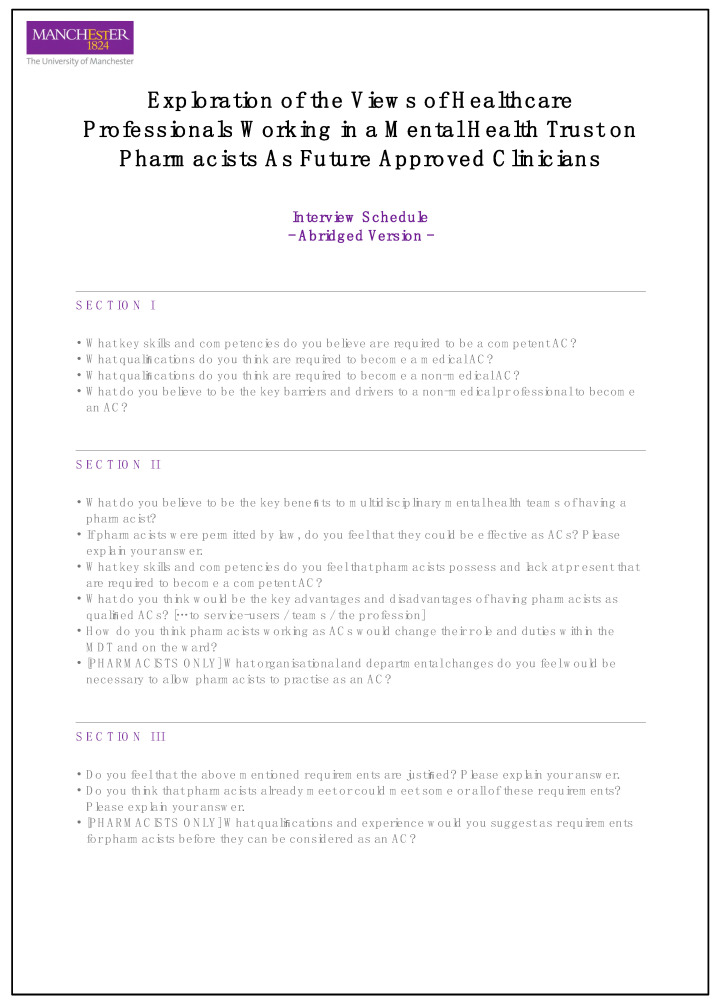
Interview schedule (abridged).

The interview schedule had been prepared by B.A. and reviewed by R.N.K. and was used flexibly during the interview process, thereby allowing each participant to share their opinions freely on all of the topics discussed. The specific questions used in the interview schedule emerged from the study aim and objectives and underwent initial pilot testing. Following completion of the first three interviews, the research team reviewed progress and determined that no changes were required to the interview schedule. Interviewees were encouraged to reflect on the perceived benefits, current status and practical utilisation of non-medical ACs before considering the possibility of mental health pharmacists being enabled—via a legislative change—to qualify and practise as ACs in the future. They were asked for their opinions on the advantages and disadvantages of pharmacists hypothetically assuming this role; to name and dissect the various discrepancies between the skills required by a pharmacist and an AC, and finally, to propose ways to bridge the gap dividing these, as well as to share their thoughts and to put forward suggestions on ways in which pharmacists could and should develop further. The pharmacists were asked additional questions in order to learn their opinions about how organisations and departments could potentially facilitate pharmacists transitioning into the non-medical AC role in future. Please refer to Figure A1 for the interview schedule (abridged).

### 2.3. Data Analysis

Each interview was audio recorded using trust-approved equipment and corresponding verbatim transcripts were produced by B.A. using the application Pages by Apple. In order to ensure study rigour was embedded in all qualitative procedures, the measures of credibility, dependability, confirmability and transferability were wilfully observed by the authors. The transcripts were analysed by B.A. strictly adhering to the six-phase thematic analysis methodology laid out by Braun and Clarke as follows: familiarising yourself with your data, generating initial codes, searching for themes, reviewing themes, defining and naming themes, and producing the report [24]. Transcripts were not checked by the participants themselves; R.N.K. reviewed six transcripts to confirm the coding framework. Responses to each interview question were initially coded using succinctly worded “labels” which served to be meaningful representations of the answers given. These were then arranged, and common patterns in responses were grouped together to form an early set of themes. The original themes were continually reviewed and adjusted as necessary in order to describe the dataset with increasing precision before the final report was generated.

## 3. Results

Following the above-detailed sampling framework and adhering to principles of data saturation and reflexive analysis, a total of 13 interviews were conducted. The mean interview duration was one hour, with the shortest interview being 53 minutes and the longest 74 minutes. None of the candidates approached refused to take part in the study and no participants dropped out. The participants included six mental health pharmacists, five medical ACs (four consultant psychiatrists and one associate specialist) and two mental health nurses (of which one was currently working towards becoming an AC and the other being a nurse consultant). The level of experience working in the field of mental health varied widely among the participants: from 2.5 to 37 years.

Opinions expressed by the participants were similar across the three professional groups and each was able to list several facilitators and barriers to mental health pharmacists potentially acting as ACs in the future. The themes generated through analysis are detailed in the following sections.

### 3.1. The Challenges Surrounding the Non-Medical AC Role

As discussed above, a brief evaluation of the wider non-medical AC role was a secondary objective of this research. Nonetheless, this topic allowed the participants to reflect on some of the drivers and barriers which may apply to mental health pharmacists potentially being considered for the AC role in future. Both drivers and barriers as identified by the participants were categorised based on whether the forces behind them were external or internal to the individuals already employed as ACs or working towards the qualification.

#### 3.1.1. Barriers

The view that there was an apparent scarcity of non-medical ACs—despite a change in the law over a decade ago allowing non-medical professionals to assume this role—was shared amongst all of the participants. Many confessed that they had never in fact encountered a non-medical AC throughout their professional career. This particular observation paved the way for the participants to begin to contemplate some of the barriers behind this, generating a total of 10 broad yet distinct themes.

Of these, one key thread revolved around the current system and status quo, which was perceived by the participants as the principal limiting factor to the uptake of the non-medical AC role.

“The traditional AC is a psychiatrist … and I imagine there may be some resistance, maybe from psychiatrists that don’t want, potentially, nurses or occupational therapists or anyone to be in that role”

(Nurse 01)

Although not the primary objective of this work, emerging themes and associated verbatim participant quotes discussing the non-medical AC role are detailed separately from the main text in Table A1.

#### 3.1.2. Drivers

Although drawbacks and complications to becoming non-medical ACs featured more heavily in participants’ responses, the discussions also generated a number of common themes with regard to drivers.

The internal drivers identified by the participants revolved around the benefits of the AC qualification itself. These consisted of the following four self-explanatory themes: increased autonomy and seniority; being more respected by colleagues; notions around career progression, such as clinical development and pay increase, and ultimately, job satisfaction.

“I think professional autonomy and job satisfaction are probably the two biggest drivers for a lot of us, isn’t it?”

(Psychiatrist 03)

The external drivers identified by the participants were found to be concentrated around three common concepts: a perceived shortage of medical staff, economic benefits of employing non-medial ACs and a progressive working model and mental health service provision.

“Our mental health services are traditionally structured with a consultant in the team generally taking the lead and being the lead for all the people under the care of that—either ward or community—team. So, increasing the scope for other professionals, actually, to allow a bit more for restructuring mental health services potentially.”

(Pharmacist 05)

### 3.2. The Role and Benefits of Mental Health Pharmacists

Interviewees were asked to reflect on the role of mental health pharmacists, in particular, on their potential benefits to the wider multidisciplinary teams. Seven distinct self-explanatory themes were generated from the dataset as follows: specific knowledge of medication; clinical knowledge and evidence-based practice; ability to prescribe (dependent upon an additional qualification); medicines reconciliation and interface working with GPs and other primary care colleagues; education and training responsibilities; medication counselling role; and provision of a safety net to colleagues.

Almost all participants regarded the pharmacists’ clinical knowledge, particularly their expertise in medication as one of the most well-known and useful benefits:

“Well, you [pharmacists] are a font of all knowledge! You know more about drugs than me, you’ve got it off the top of your head more than me… You know the interactions, you know the monitoring requirements … You’ve got that knowledge and you know how to lay your hands on knowledge that I don’t. So, yes, it’s useful to have you in the team.”

(Psychiatrist 01)

In addition to acknowledging the highly regarded educational services that pharmacists ordinarily delivered to both colleagues and service users on psychotropic medications, several participants volunteered that pharmacists not only contributed a different perspective to patient care—on occasion even challenging clinical decisions—but they particularly valued their unique viewpoint, which was not only seen as grounded in evidence-based practice but one which often provided fertile ground for clinical discussions and improved treatment for patients:

“I’m very much in favour of mental health pharmacists as part of MDTs, and particularly, in the inpatient setting. My opinion is that every MDT and every ward should have its own mental health pharmacist and, again, my opinion would be that ideally the mental health pharmacist should form part of every ward round … The ability to have a discussion between prescriber and pharmacist, I think, it really adds as a sum of a force multiplier, in terms of the effectiveness of the prescribing as well as increasing safety.”

(Psychiatrist 02)

Lastly, there was an acknowledgement, shared by many, of the unique position of the pharmacist—compared to others within the MDT—with regards to supporting service users, too:

“Also, patients tend to be a lot more honest with us from what I’ve seen … patients might open up to a pharmacist because they don’t see us in the same way as they might see the consultant; that we are often seen as a problem-solvers rather than problem-makers by the patient.”

(Pharmacist 03)

### 3.3. Skills and Competencies of Mental Health Pharmacists

Several of the interviewees were able to draw on their experiences of working with pharmacists to deduce key skills and competencies relevant to those of the AC. Most participants argued that pharmacists shared a number of skills and competencies required by an AC, albeit at a lower perceived level. These included clinical knowledge relating to pharmacological treatments, leadership and MDT working, as well as up-to-date knowledge of policies and relevant parts of the legal framework:

“Pharmacists also have got a knowledge of the Mental Health Act, in terms of different sections and that kind of thing, which would apply very well to being an RC [or an AC].”

(Psychiatrist 04)

There were several important points that both pharmacists and non-pharmacists made with regards to pharmacists not having enough experience in undertaking assessments and managing care plans:

“Assessing risks—and this is something that if you have not had experience with this, it may be difficult to master in a short period of time. It requires seeing many cases, following them up, seeing what happened with them… to understand why, when we say this person is at risk of harming themselves, why we say that. And it’s not just about doing a one-off assessment.”

(Psychiatrist 05)

These views were generally shared by the pharmacists, too:

“I don’t think we have the skills in—I think—diagnosis … I’d be inclined to say that given that doctors go through—is it six years specialist training to become a psychiatrist?—I am not sure that this is something that pharmacist should or could be doing, actually.”

(Pharmacist 01)

A few, in contrast, whilst recognising the fundamental differences between pharmacists and the other groups, were also keen to stress the similarities in terms of the common challenges faced by different professionals working towards the AC qualification:

“So, what I’m saying is if psychologists can do it—they won’t have had a vast amount of training in their undergraduate qualification on diagnosis—because they are more into therapeutic interventions—and that’s probably the same for pharmacists—so there is a bit of a gap but they’re all in the same place, aren’t they… Nurses—well, they won’t have been used to making diagnoses … and they’ve had to learn that on their job through experience—so, none of these things are insurmountable.”

(Pharmacist 05)

There were concerns expressed across professional groups about the apparent lack of sufficient patient interactions pharmacists might have in their day-to-day work. Pharmacy was commonly viewed as a predominantly “background function” which was thought to be a significant contributor to this perceived shortage of relevant skills (e.g., in-depth clinical, diagnostic and assessment skills), practical experience and on-the-job training opportunities:

“On the negative side, I don’t think pharmacists interact with patients one-to-one as much as doctors, nurses or psychologists do … For me, what is lacking is that sort of patient contact; a diagnostic curiosity.”

(Psychiatrist 03)

Many explained that this was in part caused by a discernible shortfall of mental health pharmacists, and some criticised the conventional organisational structures within mental health trusts for the way pharmacists were seemingly continuing to be utilised:

“It’s important that pharmacists are part of a team—but, of course, if they are stretched and have to visit five more wards then they’re not able to be part of the team … And this does limit their ability.”

(Psychiatrist 03)

### 3.4. Pharmacists and ACs—Bridging the Gap

There were several proposals made towards enabling pharmacists to take on increasingly clinical roles within mental health in order to start acquiring those requisite skills and competencies; some of these plans involved exploring hitherto uncharted territories and ideas about creating innovative roles, primarily through embedding pharmacists into existing multidisciplinary inpatient and community teams:

“Get them more involved in the care planning and in the assessment process… so, maybe there can be a role that is created that incorporates both and then they could build upon that pathway for the approved clinician bit.”

(Nurse 01)

All of the pharmacists interviewed, when asked to proffer solutions to achieve some of the key AC-specific competencies, were united in their response to the campaign for better integration into clinical teams:

“I guess, department-wise, you would have to have a dedicated pharmacist part of the ward.”

(Pharmacist 05)

“I think they [pharmacists] would [have to] be a lot more integrated into a team; they would probably have a bit of responsibility of managing other people and they would certainly have a clinical leadership role within the team and they would clinically be leading a multidisciplinary team.”

(Pharmacist 06)

Recognising the considerable challenges and pronounced obstacles in the way of pharmacists moving towards becoming ACs in future, some chose to abandon the idea altogether and instead proceeded to advocate that employers should be exploiting already existing formal development frameworks offered by professional bodies.

“There may well be things like credentialing or more advanced practice—so whether through a formal process or through using or reflecting on things like the advanced pharmacy practice framework, or the multi professional advanced clinical practitioner framework, or the College of Mental Health Pharmacy credentialing—this, sort of, almost helps assure people that you do practise at a certain level; and it would help assure you, I think, as well.”

(Pharmacist 05)

As a means of professional development for pharmacists to obtain skills required by an AC, some of the participants focused on formal certifications exclusively, including non-medical prescribing and advanced clinical practice; whilst others concentrated on a combination of relevant qualifications alongside various means of demonstrating competencies on the job.

“I think pharmacists meet many of the requirements for being responsible clinician as it is… but they would have to have a job that is set up for development - where people are going to have to spend maybe two years on getting clinical cases, and also maybe do a kind of an exam on top of some kind of a Masters of Psychiatry or similar plus clinical cases—a portfolio of cases, and that kind of thing. So, it’s doable but it would need to be organised.”

(Psychiatrist 04)

Almost all of the participants, however, agreed that any proposed change to improve the clinical development opportunities of mental health pharmacists would likely be a gradual process, one that would typically involve creating a unified and standardised training approach within mental health pharmacy—in particular, one that is recognised across organisations—whilst also acknowledging that this new pathway may or may not eventually lead to the enablement of the approved clinician role for pharmacists in future.

## 4. Discussion

### 4.1. Statement of Key Findings

This qualitative research explored the views and attitudes of healthcare professionals towards the role of the mental health pharmacist, and whether they could and/or should be enabled, via a legislative change, to assume the role of AC in the future; with a secondary focus to evaluate the wider non-medical AC role in its current state. This is the first study of its kind to draw comparisons between the increasingly clinically trained mental health pharmacists and non-medical approved clinicians; the juxtaposition of these professional roles is entirely novel in this field of research.

Several participants proposed that in order to legally and practically enable pharmacists to act as ACs in future, organisations must first support them by offering ample opportunities to obtain and demonstrate the application of the necessary skills and competencies required of ACs. Many expressed that this would only be likely to happen if pharmacists were fully incorporated into clinical teams, working away from the busy dispensary environment. Some proffered possible solutions such as creating novel clinical roles and others highlighted the importance of developing nationally recognised training pathways.

### 4.2. Strengths and Weaknesses

Key strengths of this research include the exploration of a novel subject area, as well as the attainment of data saturation in this cohort of participants.

Conversely, several significant limitations have been identified. Firstly, there were no qualified non-medical ACs employed at the participating trust to be recruited to this study; their accounts could potentially have introduced unique insights not expressed by the other participants. Similarly, the inclusion of a wider range of relevant professional groups, such as clinical psychologists, occupational therapists and social workers, from multiple different settings and organisations, may have generated additional themes on the subject. Furthermore, the scope of this study was restricted by resource limitations, which ultimately informed the relatively small sample size from a single institution. Lastly, some of the participants shared a close working relationship with and belonged to the same professional network as the author, B.A.; though this was mitigated by explicitly explaining to each interviewee the importance of providing honest and impartial responses to questions, the risk of acquiescence bias cannot be ruled out.

### 4.3. Interpretation

Participants in this research provided insights and opinions on the apparent limited wider uptake of the non-AC role over a decade after its introduction. It is evident from the participants’ responses that fundamental changes may be required to increase and in turn sustain the number of non-medical ACs. A standard approach to allow all permitted groups within the legislative framework, irrespective of their professional affiliation, to become ACs may facilitate this goal. In addition to being able to qualify via the portfolio route, applicants could also be given the opportunity to undertake a dedicated training pathway, one that is—in some aspects—akin to the clinical developmental roadmap developed for psychiatric trainees. People choosing to continue to proceed via the portfolio route, however, must also be supported adequately by their respective employers—a common key concern highlighted in this study as well as in the works of Ebrahim and Oates et al. [4,5]. To enable this there must be sufficient funding made available to NHS trusts to create training posts as well as new non-medical ACs roles for those already qualified. In addition, the creation of more specialist posts—specifically for non-medical ACs may also be worth exploring, as suggested by some of the participants—whether through a local pilot scheme or as part of a nationwide endeavour—where non-medical ACs may safely be allowed to make certain autonomous decisions without having to rely on the support of medical colleagues.

Discussions about mental health pharmacists specifically, generated a number of salient themes, including strengths and areas of development in their current role and scope of practice, wider contributions to patient care as well as their unexploited potential. As reported in the literature, the under-utilisation of mental health pharmacists remains commonplace [25], despite there having been several recommendations made by key stakeholders and bodies on how to facilitate the transformation of pharmacists into advanced roles [26,27]. Specifically, The NHS Long Term Plan also highlights the necessity of up-skilling mental health pharmacists and promoting their wider deployment across services [28]. The particular recommendation by participants of this research to fully embed pharmacists into multidisciplinary clinical teams corroborates existing findings on the topic [13]. There are already significant changes being made to the standards for the undergraduate curriculum of pharmacists, enabling all in future to independently prescribe from the point of registration [29]. This is expected to narrow the gap, to an extent, in pharmacists’ competencies highlighted in this study, such as in diagnosis and assessments. Participants also highlighted the need for changes to pharmacists’ day-to-day roles and responsibilities so as to provide them with sufficient relevant experience. In addition to the requirement to update current legislation to legally allow pharmacists to train to be an AC, there were also suggestions for the creation of bespoke training pathways to equip pharmacists with the required knowledge, skills and competencies, particularly in the domains of care planning, leadership and multidisciplinary working, diagnosis and assessments—something which could be incorporated further into future accreditation standards of specialist mental health pharmacists. Presently, several organisations require mental health pharmacists to complete specific postgraduate courses, and there also exists the opportunity to further demonstrate expertise in the field of mental health pharmacy through credentialed membership of the UK College of Mental Health Pharmacy. Naturally, the central focus of these is pharmacotherapy, and whilst there is some overlap, these are not designed specifically to meet the knowledge, skill and competency requirements of an AC. In order to attain these, pharmacists may be required to undertake additional training, thus becoming independent prescribers and/or advanced practitioners. The latter is currently receiving renewed attention following the recent release of the first national curriculum and capabilities framework for mental health advanced practice by Health Education England, which appears to map capabilities most closely to those required by an AC [30]. Nonetheless, to date, there does not exist a clear single unified framework, credential or qualification available to mental health pharmacists to demonstrate capability as there does for the professionals presently enabled by the MHA to train to be ACs.

### 4.4. Further Research

This study has shown that there is scope to carry out further research to gain a better understanding of the challenges facing the clinical development and enhanced utilisation of highly specialised mental health pharmacists across services. In particular, the findings of in-depth interviews and focus groups could inform subsequent national surveys and be valuable to deepening the understanding of this subject and it is recommended that these works be conducted with professionals from various geographical areas, and including mental health pharmacists delivering patient-facing clinics as well as those in consultant and other relevant leadership posts. Additional studies exploring the views of individuals involved in making decisions about the MHA (such as those currently working on the white paper “Reforming the Mental Health Act”) may also provide further insights and facilitate progress towards mental health pharmacists being permitted to become non-medical approved clinicians in the future.

With respect to the non-medical AC role, further qualitative research, specifically involving qualified non-medical ACs, is recommended in order to better understand the challenges faced by this group of individuals. Quantitative studies conducted with practising non-medical ACs, including those in specialist recovery-oriented settings, will also help evaluate the impact of the non-medical AC role and inform improved and targeted service development in future.

## 5. Conclusions

This work explored collegiate views about two currently distinct professional roles - primarily that of the mental health pharmacist, and secondarily that of the non-medical AC—not only discussing their current status and unexploited potentials but also attempting to seek opinions on how they could be further transformed, enhanced and better utilised. This research has found that changes to the skill mix within multidisciplinary mental health teams as well as to the training of staff may be required to equip pharmacists with essential skills to be able to begin to transition towards the AC role. These goals may be supported by further research to understand the development and utilisation of highly specialist mental health pharmacists. This effort could be bolstered by mental health organisations making better use of the advanced clinical skills of their pharmacists and actively facilitating their integration into multidisciplinary teams. Additionally, multi-professional leadership organisations could work together to create a nationally recognised and unified credentialing framework for mental health pharmacists encompassing the skills and competencies required by approved clinicians under the Mental Health Act, whilst also ensuring that the current make-up of multidisciplinary teams reflects the breadth of professionals involved in patient care.

## Data Availability

The data presented in this study are available on request from the corresponding author. The data are not publicly available due to ethical reasons.

## Appendix A

Box A1 Summary of key differences between medical and non-medical ACs.
**Routes of Qualifying**
Non-medical professionals (as well as medical doctors who are not on the GMC Specialist Register in Psychiatry) wishing to become an AC may only qualify via the portfolio route. This is an extensive process; one that is “grounded in a critical engagement with human rights-based interpretations of the law” [4]. In order to achieve AC status, psychiatrists, however, have the opportunity to forego the portfolio route and complete a distinctly shorter medico-legal induction course (lasting for as little as two days), whilst also automatically receiving section 12 approval—an additional set of powers under the MHA [31].
**Legal Powers**
There are also notable differences between the powers of medical and non-medical ACs. For instance, only a medical AC can provide medical recommendations for guardianship as well as for Sections 2–4 under the MHA, and only they are permitted to determine the capacity to consent to treatment and to authorise emergency medical treatment. Furthermore, only a medical AC—when acting as a responsible clinician jointly with a second medical doctor—can initiate detention under the MHA. Fennell attributes these inconsistencies to fundamental differences in the clinical training of physicians and non-medical professionals, reasoning that only “doctors with the broad diagnostic skills should decide whether people should be detained” [32].

**Table A1:** Key themes on the barriers for non-medical ACs as identified by participants.

BARRIERS—INTERNAL	
**Theme**	**Description with Participant Quote**
1 Commitment	One explanation by a large number of the participants for the relative lack of practising non-medical ACs was that albeit adequately skilled and experienced, many individuals would likely be put off by the amount of work required to eventually qualify as ACs via the portfolio route. What is more, many believed that once one had embarked on this training pathway, there was seldom sufficient continuing support from organisations—for instance, protected time for learning—which meant that a great proportion would eventually completely abandon their training. “You know, it’s very hard work to get there—I think most people will drop at the first or second hurdle. Because you’ve got your own job to do, so how are you going to find the time to skill yourself up with all this stuff you need?” (Psychiatrist 01)
2 Renumeration	Participants shared doubts about the standardisation of the pay structure for newly qualified non-medical ACs. Many showed concerns that due to the perceived lack of qualified non-medical ACs, each organisation might choose to approach remuneration differently and they also expressed scepticism about there being a consistent and fair strategy across the board with regards to pay progression down the line. “Is there a clear pathway to it [practising as a non-medical AC]? And if there is, is there any extra reward as well as responsibility? So, is it associated with a higher banding, and all of that.” (Pharmacist 06)
3 Responsibility and Accountability	Participants reported that many professionals considering training to be an AC might be demoralised by the amount of responsibility and accountability associated with the role. Furthermore, several participants had doubts about the provision of appropriate and sufficient indemnity by employing trusts. “This is a big thing; you have got a lot of power and influence if you’re the AC, for someone you can make decisions on their behalf, so, this is something that could be quite scary for a lot of people.” (Pharmacist 03)
4 Therapeutic Relationship	Participants felt that a number of non-medical professionals working in mental health greatly valued the therapeutic relationship they established with service users. Some, therefore, feared that, by assuming the role of the AC—which includes having to apply coercive powers under the MHA—one would have to sacrifice this highly rewarding aspect of their day-to-day job. “I see it as, kind of, a barrier, thinking, why would I want to be [the responsible clinician]—let’s leave the detention to somebody else and I’m the one who does the therapeutic interventions.” (Pharmacist 06)
5 Additional Jobs	Many expressed the suspicion that even after qualifying as an AC professionals might still be required to carry on working in their current substantive role for a period of time due to the assumption that there might be a greater demand for a job role that is established and widely recognised in the system—one that could be deemed by employers to be too valuable to lose—compared with one that they would likely be unfamiliar with. “So, I suspect once someone qualifies and wants to practise as an AC or an RC they would keep their current role and responsibilities and they would add the extras on to that—that’s how things work within the NHS!” (Pharmacist 01)
6 Inferiority	Some participants stated that despite the fact that some non-medical ACs might also act as non-medical prescribers, some might not possess the experience and expertise to be able to prescribe completely independently, and thus might risk being regarded as inferior compared to their medical counterparts. Some felt that this was one of the fundamental issues with the non-medical AC role, too. “Certainly, I have had that with prescribing: some people don’t believe that nurses should be prescribers, and I think there is probably an element of that with ACs as well.” (Nurse 01)
**BARRIERS—EXTERNAL**	
**Theme**	**Description with Participant Quote**
1 Awareness	Some participants stated that the concept of the non-medical AC had never truly materialised and thus the role was not frequently spoken of by organisations. They argued that as the non-medical AC role was less known amongst potential candidates, it meant that very few would even consider it as a route of career progression and organisations were also unlikely to be able to provide much information to anyone even wishing to find out more about the role. “The barrier is the story around it, and the communication around it … I suppose the whole framework around it is kind of not clear to me; and there is always advertising around, you know, “Do this non-medical prescribing course or the advanced practitioner role!”, but the publicity around responsible clinician is quite small.” (Pharmacist 06)
2 Training Gap	Several participants stated that unlike their medical counterparts non-medical ACs did not have a clear training pathway and that they were only able to qualify via the portfolio route. They explained that this route was a lengthy process during which the employing trust was often required to support trainees by providing additional protected time whilst also needing to manage the competing operational demands of the service. Many indicated that without a dedicated training pathway the uptake of the non-medical AC role would be unlikely to change. “I think perhaps barriers would be in terms of knowledge. There is doctrine—there is a fairly established path for psychiatrists that they’re expected to progress in order to become ACs. Whilst not absolutely compulsory, it is seen as the norm; it is very commonly done as a clear established path … My perception is that for non-medical ACs those well-trodden pathways, as it were, don’t exist; there is perhaps not such a well-defined path or at least one where a non-medic that is becoming an AC is perhaps less likely to have other colleagues who’ve been along that route to help them along that path—it may be less established.” (Psychiatrist 02)
3 Status Quo	Several participants reflected that prior to the 2007 amendment only medical doctors were in possession of the AC powers under the Mental Health Act. Some believed that despite the change in law, there was still a need for a substantial shift in the culture and system of mental health provision in England and Wales, adding that there might still remain resistance—by colleagues and service users alike—toward fully accepting non-medical professionals as ACs. “I don’t understand why a pharmacist or anybody else would want to be an AC unless we all change our mindset as to what an AC is … I think it’s too big a leap for 2020.” (Psychiatrist 01)
4 Impracticalities	Many argued that an important reason for the poor uptake of the non-medical AC role was that non-medical professionals were not able to completely replace medical ACs due to fundamental differences in their skills as well as in some of the powers they could wield under the MHA. They reasoned that this rendered the non-medical AC role highly impractical. “If you could have an RC who is a non-medic, you would have to have an AC—a medic—working with you. Because, for instance, a psychologist working as an RC may not know much about medications or can’t prescribe, and they will still need someone to prescribe. So, then they would have an AC who is a medical doctor working with them. Now, I’m not sure there are many ACs who would want to do that role. Because they’ll say ‘If I’m going to do this, I might as well be the RC.’” (Psychiatrist 03)
